# Supplementary material for: SCC3 is an axial element essential for homologous chromosome pairing and synapsis
Source: eLife. 2024 Jun 12;13:RP94180. doi: 10.7554/eLife.94180 (PMC11168746; doi:10.7554/eLife.94180)
Supplement: Supplementary file 1. [file elife-94180-supp1.docx]

**Supplementary table 1.** Primers for Real-time PCR and plasmid construction

| Primer name | | Primer sequence | Description |
| --- | --- | --- | --- |
| SCC3-Cas9-F | GGCAATTGCGCGAGGCACCAACAT | | CRISPR-Cas9 |
| SCC3-Cas9-R | AAACATGTTGGTGCCTCGCGCAAT | |  |
| REC8-Cas9-F | GGCAGAGCCTTCCGCGCGAGGAGC | | CRISPR-Cas9 |
| REC8-Cas9-R | AAACGCTCCTCGCGCGGAAGGCTC | |  |
| SMC1-RT-F | TAAGGGGACGCAGACAATCG | | Real-time PCR |
| SMC1-RT-R | GGGCGTAGATGAGGTCCTTG | |  |
| SMC3-RT-F | CGTTGTGATCCAGCCCCATT | | Real-time PCR |
| SMC3-RT-R | CTGGTCGGAATGTCGTTGCT | |  |
| SCC1-RT-F | ATGTTCTGTCCGCAATGGGT | | Real-time PCR |
| SCC1-RT-R | TGTCAACACCCTGAATCGCA | |  |
| SCC3-RT-F | GTTATTCATGCACCGCTATCG | | Real-time PCR |
| SCC3-RT-R | CAGGCTTTGCAAGGCAAGAA | |  |
| REC8-RT-F | TCCACTCGTACCTCAAGCTA | | Real-time PCR |
| REC8-RT-R | GTTGCTAAAACGCATGCTTG | |  |
| PAIR2-RT-F | GATCGACGATGGGTTGCCT | | Real-time PCR |
| PAIR2-RT-R | AACAAACGGACGCCTGCTC | |  |
| PAIR3-RT-F | CAAGAAGAGAACAGGACAGT | | Real-time PCR |
| PAIR3-RT-R | GTAGAACGATGAGTAGACAATG | |  |
| SGO1-RT-F | TCAATCAGCTGTGCCATCTT | | Real-time PCR |
| SGO1-RT-R | CATCTTGCCACCACA AATCA | |  |
| ZEP1-RT-F | CAGCAGGATAATGAGCATAA | | Real-time PCR |
| ZEP1-RT-R | GGTCTCAGGACTAACCAACT | |  |
| SCC3-AD-F | GACGTACCAGATTACGCTCATATGGACGAGACCCTAGCCTCC | | Constructing vectors for Y2H experiments |
| SCC3-AD-R | TCTGCAGCTCGAGCTCGATGTCAGCTATTGCTTCCTGATGCCCT | |  |
| SCC3-BD-F | ATCTCAGAGGAGGACCTGCATATGGACGAGACCCTAGCCTCC | | Constructing vectors for Y2H experiments |
| SCC3-BD-R | GCGGCCGCTGCAGGTCGACGTCAGCTATTGCTTCCTGATGCCCT | |  |
| SCC1-AD-F | GACGTACCAGATTACGCTCATATGTTCTACTCGCAGTTCATC | | Constructing vectors for Y2H experiments |
| SCC1-AD-R | TCTGCAGCTCGAGCTCGATGTCAGAAATCTGACTTCAGGAGCTT | |  |
| SCC1-BD-F | ATCTCAGAGGAGGACCTGCATATGTTCTACTCGCAGTTCATC | | Constructing vectors for Y2H experiments |
| SCC1-BD-R | GCGGCCGCTGCAGGTCGACGTCAGAAATCTGACTTCAGGAGCTT | |  |
| SMC1-AD-F | GACGTACCAGATTACGCTCATATGGCCGCGGCGGCGGCAGG | | Constructing vectors for Y2H experiments |
| SMC1-AD-R | TCTGCAGCTCGAGCTCGATGTCACAAAAGAGGTACTTTAA | |  |
| SMC1-BD-F | ATCTCAGAGGAGGACCTGCATATGGCCGCGGCGGCGGCAGG | | Constructing vectors for Y2H experiments |
| SMC1-BD-R | GCGGCCGCTGCAGGTCGACGTCACAAAAGAGGTACTTTAA | |  |
| SMC3-AD-F | GACGTACCAGATTACGCTCATATGGATGCTGAGCGTGACCA | | Constructing vectors for Y2H experiments |
| SMC3-AD-R | TCTGCAGCTCGAGCTCGATGTCAGCTAGCGTTGTGTGTCT | |  |
| SMC3-BD-F | ATCTCAGAGGAGGACCTGCATATGGATGCTGAGCGTGACCA | | Constructing vectors for Y2H experiments |
| SMC3-BD-R | GCGGCCGCTGCAGGTCGACGTCAGCTAGCGTTGTGTGTCT | |  |
| REC8-BD-F | ATCTCAGAGGAGGACCTGCATATGTTCTACTCGCACCAGC | | Constructing vectors for Y2H experiments |
| REC8-BD-R | GCGGCCGCTGCAGGTCGACGCATCTTTGGTCCCCTCGAGAT | |  |
| SCC3-NE/CE-F | CCCAGGCCTACTAGTGGATCCATGGACGAGACCCTAGCCTCC | | Constructing vectors for BiFC experiments |
| SCC3-NE/CE-R | CCCGGGAGCGGTACCCTCGAGTCAGCTATTGCTTCCTGATGCCCT | |  |
| REC8-NE/CE-CF | CCCAGGCCTACTAGTGGATCCATGTTCTACTCGCACCAGC | | Constructing vectors for BiFC experiments |
| REC8-NE/CE-R | ATCCCGGGAGCGGTACCCTCCATCTTTGGTCCCCTCGAGAT | |  |
| SCC3-Ab-F | GACGACGACGACAAGGCCATGCATGTAAGTGATGGGGAAAA | | Antibody production |
| SCC3-Ab-R | GCAGCCGGATCTCAGTGGTGGGCTATTGCTTCCTGATGCCC | |  |
